# Non-Fatal Injury in Thailand From 2005 to 2013: Incidence Trends and Links to Alcohol Consumption Patterns in the Thai Cohort Study

**DOI:** 10.2188/jea.JE20150218

**Published:** 2016-09-05

**Authors:** Mami Wakabayashi, Janneke Berecki-Gisolf, Cathy Banwell, Matthew Kelly, Vasoontara Yiengprugsawan, Rebecca McKetin, Sam-ang Seubsman, Hiroyasu Iso, Adrian Sleigh, the Thai Cohort Study Team

**Affiliations:** 1 Public Health, Department of Social Medicine, Osaka University Graduate School of Medicine, Suita, Osaka, Japan; 1 大阪大学大学院, 医学系研究科, 社会環境医学講座, 公衆衛生学, 日本; 2 National Centre for Epidemiology and Population Health, Research School of Population Health, Australian National University, Australia; 2 オーストラリア国立大学, 公衆衛生大学院,国立疫学公衆衛生センター, オーストラリア; 3 Monash Injury Research Institute, Monash University, Melbourne, Australia; 3 モナッシュ大学, メルボルン校, モナッシュ外傷研究所, オーストラリア; 4 Centre for Research on Ageing, Health and Wellbeing, Research School of Population Health, Australian National University, Australia; 4 オーストラリア国立大学, 公衆衛生大学院,高齢化＆健康福祉研究所, オーストラリア; 5 School of Human Ecology, The Sukhothai Thammathirat Open University, Thailand; 5 スコタイタマティラート通信教育大学, 人間生態学大学院, タイ

**Keywords:** Thailand, injury, alcohol, health transition, socio-economic status, タイ, 傷害, 飲酒, 健康移り変わり, 社会経済的地位

## Abstract

**Background:**

We analyzed population-based injury trends and the association between injury and alcohol consumption patterns in Thailand, a middle-income country undergoing rapid social change.

**Methods:**

A nationwide cohort of 42 785 Thai adult Open University students, who were aged 15 to 87 years at enrolment, participated in cross-sectional assessments at baseline (2005) and 8 years later (2013). Incident non-fatal traffic and non-traffic injuries were recorded. Alcohol consumption patterns were categorized as follows: non-drinkers, occasional light drinkers, occasional heavy drinkers, regular drinkers, and ex-drinkers. Logistic regression was used to assess associations in 2005 and 2013 between injuries and alcohol consumption. We adjusted odds ratios (ORs) for socio-demographic factors, stress, health behaviors, and risk-taking behaviors.

**Results:**

Incidence estimates in 2013 were standardized to the age structure of 2005: the standardized rates were 10% (95% confidence interval [CI], 9.32–9.89) for participants with at least one non-traffic injury and 5% (95% CI, 4.86–5.29) for those with at least one traffic injury. Both standardized incidences for non-traffic and traffic injuries were significantly lower than corresponding rates in 2005 (20% and 6%, respectively). Alcohol consumption was significantly associated with non-traffic injury in 2005, but the association disappeared in 2013. For example, non-traffic injury was associated with regular drinking (adjusted OR 1.17; 95% CI, 1.01–1.40) in 2005, but not in 2013 (adjusted OR 0.89; 95% CI, 0.73–1.10). In both survey years, traffic injury was not associated with occasional heavy drinking when adjusted for health and risk-taking behavior.

**Conclusions:**

We examined non-fatal injury and the health-risk transition in Thailand in 2005 and 2013. Our data revealed decreases in alcohol consumption and non-fatal injury in the Thai Cohort between 2005 and 2013. Alcohol-related injury in Thailand today could be amenable to preventive intervention.

## 抄録

**背景：:**

急速な社会的変化を遂げている中所得国であるタイにおける地域住民を対象とした外傷の発生動向と飲酒形態との関連について分析する。

**方法：:**

通信教育大学に通うタイ全土の社会人学生42,785名（2005年時点で15-87歳）が2005年と8年後の2013年の両年共の横断調査に参加した。非致死的外傷である、交通事故による外傷とそれ以外の外傷を記録した。飲酒形態は、非飲酒者、軽度機会飲酒者、過度機会飲酒者、習慣飲酒者、過去飲酒者に分けた。2005年と2013年における各々の外傷と飲酒形態との関係について社会経済的要因、ストレス、健康行動、危険選択性向を調整しロジスティック回帰分析により解析した。

**結果：:**

2005年の年齢構成で調整された2013年の非交通事故外傷の発症率は10％（95%信頼区間9.32-9.89）、交通事故外傷の発症率は5％(95%信頼区間4.86-5.29)であった。それぞれの年齢調整外傷発生率は2005年と比較し統計的に有意に低かった（2005年はそれぞれ20％と6％）。

2005年時点で、飲酒習慣は非交通事故外傷と統計的に有意な関連が見られたが、2013年に関連は見られなかった。例えば、習慣飲酒者と非交通事故外傷は2005年時点では関連した（調整オッズ比1.17, 95%信頼区間1.01-1.40）が、2013年時点では関連はなかった（調整オッズ比0.89,95%信頼区間0.73-1.10）。過度機会飲酒者や習慣飲酒者と交通事故外傷とは、両年の研究ともに健康行動と危険選択性向を調整したところ、関連性は認められなかった。

**結論：:**

タイでの2005年と2013年における非致死的外傷と健康状態の移り変わりについて調査した。我々のデータから、飲酒習慣と非致死的外傷が2005年から2013年にかけて減少していることが明らかになった。外傷に関連した飲酒習慣はタイにおける予防政策に反応している可能性がある。

## INTRODUCTION

Injury in developing countries is a public health problem. In middle-income countries of Southeast Asia, including Thailand, road injury is one of the top 10 causes of death.^1^^,^^2^ In addition, alcohol consumption is broadly associated with the risk of injury. Alcohol was the number one cause of burden in disability-adjusted life years (DALYs) among men in Thailand in 2009.^3^ A major barrier to addressing the problem in low- to middle-income countries is the lack of information about injuries and related demographic, psychosocial, and personal risk-taking factors.

Alcohol consumption in Southeast Asia in 2008 was highest in Thailand, with an average annual intake per capita equivalent to 7.08 liters of pure alcohol, which was higher than the world average of 6.65 liter per capita.^4^ Unlike Western countries, few women drink in Thailand.^5^ Thai alcohol consumption steadily increased from 1990 to the early 2000s,^5^ partly due to de-restriction of alcohol sales in 1997.^6^ In 2001, the Thai Health Promotion Foundation, funded by new alcohol and tobacco taxes, began to promote smoking cessation and alcohol control.^6^ The government has responded by introducing more alcohol taxes and new restrictions on sales, increasing the drinking age to 20 years, and performing drunk-driving campaigns.^6^^,^^7^ At present, alcohol use in Thailand is declining, with a decreasing trend in new female drinkers.^6^

In developed countries, the association between acute alcohol consumption and injury has been well established.^8^^–^^11^ Alcohol use is a strong risk factor for all causes of injuries, including traffic incidents,^12^ violence,^13^ suicide,^14^ and other non-traffic injuries.^15^^–^^17^ At the population level, risk of injury increases incrementally with increasing average daily volume of alcohol intake.^18^

Here, we report our cohort study of injury trends in middle-income Thailand during a period of rapid social change. Given the relatively high alcohol consumption in Thailand and its importance to DALYs lost, we focused on connections between alcohol and injury across socio-geographic groups, noting and interpreting the changes occurring over an 8-year period. Such local data help clarify and anticipate the impact of alcohol use on injury in the Thai population.

## METHODS

### Generating the study population

We studied 42 785 members of a Thai Cohort examined in 2005 and 2013. The original Thai Cohort Study (TCS) members were recruited in 2005 from 200 000 Sukhothai Thammathirat Open University (STOU) students, community-embedded adult distance learners residing throughout Thailand. STOU is a national Open University at which any adult can enroll, qualifying with high school graduation or substantial life experience, paying low fees while taking up to 12 years to complete their degree. In 2005, a 20-page baseline questionnaire was mailed to all STOU students who had completed at least their first semester, with 87 151 (44%) responding. Details of data-processing procedures can be found elsewhere.^19^ Respondents at the 2005 baseline were similar to other STOU students in age distribution, sex ratio, marital and socio-economic status, and field of study.^20^ Also, compared to the general Thai adult population in 2005, TCS members were more educated but otherwise similar in terms of socio-economic level, national geo-demographic distribution, and religious and ethnic mix.^20^

Overall, 42 785 TCS members answered both baseline (2005) and 8-year follow-up (2013) questionnaires. For this report, we excluded those with missing responses for alcohol or injury questions (826 in 2005 and 1981 in 2013); 39 978 cohort members remained after exclusions. Proportions missing co-variable data in 2005 or 2013 were low, ranging from 0.2% and 0.5% for education to 10.8% and 9.2% for occupation in the baseline and follow-up questionnaires, respectively.

### Study design

We analyzed alcohol drinking habit and injury in the cohort at the beginning and at the end of the 8-year period of longitudinal observation, noting associations and secular trends after adjusting for potential effects of an array of socio-demographic and personal factors. We recorded injuries recalled for the year before the assessments in 2005 and 2013. This 12-month period was chosen as it was closest in time and thus subject to the least recall error.

### Measures

#### Incidence of non-fatal injuries

In 2005, participants were asked the following questions: “In the last 12 months, how many injuries have you had that were serious enough to interfere with daily activities and/or required medical treatment (‘None’, ‘one’, ‘two’, ‘three’, ‘four or more’)?”, and “Was this serious injury related to road traffic? (‘Yes’, ‘No’)”. In 2013, participants were asked two independent questions: “In the last 12 months how many times did you get injured in a traffic crash (‘None’, ‘once’, ‘twice’, ‘three times’, ‘four times or more’)?” and “In the last 12 months how many times did you have a non-traffic injury?”. Additional questions about injury severity (“When you experienced your most serious traffic or non-traffic injury, did you receive medical care and/or limit your normal activities for 1 day or more?”) in 2013 enabled us to harmonize our findings with the questionnaire in 2005. The recalled incidence of traffic injury was 2242 (6%) in 2005 and 2423 (6%) in 2013; recalled incidence of non-traffic injury was 7896 (20%) and 4866 (12%).

#### Alcohol consumption in 2005

Alcohol consumption patterns in 2005 were assessed using the following two questions: “Have you ever drunk alcohol (‘No, never’, ‘Occasional social drinker’, ‘Current regular drinker’, ‘Used to drink before, now stopped’)?”, and “How many drinks of alcohol do/did you have in one sitting when you are/were drinking (‘less than 2 drinks’, ‘2–3 drinks’, ‘4–5 drinks’, ‘6 drinks or more’)?”. These two questions replicated those used in a 2004 Thai survey of cigarette smoking and alcohol drinking and measure typical use (not available in English). Participants who answered “No, never” in the first question were classified as a “non-drinkers” (*n* = 11 299), and those who answered “Used to drink before, now stopped” were classified as “ex-drinkers” (*n* = 3411). Current drinkers were categorized into three categories: occasional drinkers who reported drinking ≥4 drinks per occasion (occasional heavy drinkers, *n* = 10 531); occasional light drinkers who drank <4 drinks per occasion (*n* = 12 733); and regular drinkers (*n* = 2004). We separated occasional drinkers by whether or not they drank ≥4 drinks on a single occasion, because this corresponds to drinking to intoxication in a Thai person^21^ and corresponds approximately to international definitions of binge drinking.^22^

#### Alcohol consumption in 2013

Alcohol consumption patterns in 2013 were assessed using the following item: “Please describe your current alcohol drinking (choose one answer and indicate amount for choices 3 and 4), with responses of (1 ‘Don’t drink’, 2 ‘Used to drink but quit’, 3 ‘Drink in social situation, about __ glasses/week’, 4 ‘Current regular drinker of about __ glasses/day’)”. Participants who answered they ‘Drink in a social situation’ were sub-divided into occasional light (≤3 glasses/week) and occasional heavy drinkers (>3 glasses/week). There were five categories of alcohol consumption in total: “non-drinker” (*n* = 18 167), “occasional light drinker” (*n* = 7610), “occasional heavy drinker” (*n* = 7314), “regular drinker” (*n* = 1666), and “ex-drinker” (*n* = 5221).

#### Demographic, mental and behavioral factors

TCS questionnaires covered an array of factors that could affect alcohol drinking or injury.^23^^,^^24^ These included demographic variables (age, sex, urban residence, income, education, occupation, and marital status) and health behavior (smoking and exercise). Life-course migration was based on participants’ current residence and residence at age 10–12 years (urban-urban, urban-country, country-urban, country-country). Urban residence in 2005 and 2013 was based on participants’ current residence. Mental distress was scored 0–12 (0 is indicating no distress) using the three anxiety-oriented items from Kessler scale (K6).^25^ Social support was scored 0–16 (0 is indicating no social support) using four questions from the Irish Social Science Data questionnaire on social capital.^26^ Risk-taking behaviors were measured by self-reported drunk-driving and usage of seatbelts and helmets.

### Statistical analysis

All analyses were performed with SAS 9.3 software (SAS Institute, Cary, NC, USA). The 2005 and 2013 incidences of injury were cross tabulated with socio-demographic, health behavior, social capital, and psychosocial stress variables, with trends for proportions and means evaluated. Since we performed cross-sectional analyses in 2005 and 2013, we used separate multivariable logistic regression models to calculate odds ratios (ORs) and 95% confidence intervals (CIs) of traffic and non-traffic injury for each drinking category relative to non-drinkers. The results were adjusted for demographic variables, health behavior, and risk-taking behavior variables. All tests were two-sided, and significance was set at *P* < 0.05. When incidence rates were directly compared, the estimates for 2013 were standardized to the age structure for 2005.

### Ethical approval

The study was approved by the Sukhothai Thammathirat Open University Research and Development Institute (protocol 0522/10) and the Australian National University Human Research Ethics Committee (protocol 2004344, 2009/570). Informed written consent was provided by all participants.

## RESULTS

### Distribution of alcohol consumption patterns in 2005 and 2013

In 2005, men frequently reported occasional heavy (45%) and regular drinking (11%). Non-drinking was much more common among women (42%) than men (11%). Drunk-driving was much more frequent among men (48%) than women (8%). By 2013, drinking among cohort members had declined; 66% of women were now non-drinkers, fewer men reported occasional heavy (34%) or regular drinking (9%), and drunk-driving had declined for both men (35%) and women (5%) (Table 1).

**Table 1.** Characteristics of Thai Cohort members (*n* = 39 978) in 2005 and 2013

		Male (*n* = 17 902)		Female (*n* = 22 076)	
	
		2005	2013^h^	2005	2013^h^
Mean (SD) age, years		34.2 (8.8)	42.2 (8.8)	30.9 (7.7)	38.8 (7.7)

Education	High school or lower	8915 (50)	2803 (16)	8401 (38)	2427 (11)
	Post-high school diploma	4029 (23)	1139 (6)	6702 (30)	1461 (7)
	University or higher	4927 (28)	13 868 (77)	6916 (31)	18 087 (82)

Income (Baht/month)^a^	≤7000	5082 (28)	1517 (8)	9010 (41)	2222 (10)
	7001–20 000	9621 (54)	7628 (43)	10 488 (48)	12 164 (55)
	≥20 000	2930 (16)	8603 (48)	2105 (10)	7456 (34)

Marital status	Not married	7537 (42)	4030 (23)	12 617 (57)	7498 (34)
	Married	9972 (56)	13 588 (76)	8971 (41)	14 250 (65)

Location	Bangkok	2487 (14)	2428 (14)	4031 (18)	3867 (18)
	Central/East	4995 (28)	5184 (29)	6795 (31)	7051 (32)
	North	3819 (21)	3820 (21)	4196 (19)	4214 (19)
	Northeast	4386 (25)	4346 (24)	4024 (18)	3999 (18)
	South	2105 (12)	2124 (12)	2923 (13)	2945 (13)

Urban residence		8772 (49)	9579 (54)	11 167 (51)	12 349 (56)
Life-course migration^b^	Rural & Rural	8153 (46)	7315 (42)	9791 (44)	8622 (40)
	Rural & City	5351 (30)	6086 (35)	6148 (28)	7190 (33)
	City & Rural	793 (4)	752 (4)	931 (4)	840 (4)
	City & City	3383 (19)	3402 (19)	4983 (23)	5044 (23)

Job	Professional/manager	5231 (29)	6823 (38)	6283 (28)	9484 (43)
	Skilled worker/manual labor	4214 (24)	3023 (17)	3468 (16)	2675 (12)
	Office assistance	4481 (25)	5243 (29)	7219 (33)	6971 (32)

Mean (SD) social support score^c^		6.7 (2.3)	7.0 (2.6)	6.8 (2.2)	7.2 (2.5)

Mean (SD) psychological distress score^d^		5.5 (2.1)	3.9 (2.2)	5.7 (2.1)	3.9 (2.2)

Exercise^e^	Mean (SD) walking, times/week	2.5 (3.4)	6.7 (9)	1.2 (2.5)	5.9 (8.2)
	Mean (SD) moderate, times/week	2.3 (3.3)	3.4 (5.1)	1.3 (2.7)	2.1 (3.7)
	Mean (SD) vigorous, times/week	4.9 (5.9)	4.4 (6.1)	4.9 (5.7)	3.8 (5.2)

Alcohol	Non-drinker	1944 (11)	3575 (20)	9355 (42)	14 592 (66)
	Occasional light drinker^f^	4204 (23)	3746 (21)	8529 (39)	3864 (18)
	Occasional heavy drinker^g^	7987 (45)	6012 (34)	2544 (12)	1302 (6)
	Regular drinker	1884 (11)	1585 (9)	120 (1)	81 (0)
	Ex-drinker	1883 (11)	2984 (17)	1528 (7)	2237 (10)

Smoking	Non-smoker	8455 (47)	14 964 (84)	20 670 (94)	21 935 (99)
	Current smoker	3081 (17)	2863 (15)	153 (1)	128 (1)

Drunk-driving	No	6587 (37)	9859 (55)	8947 (41)	17 439 (79)
	Yes	8512 (48)	6351 (35)	1687 (8)	1184 (5)

Frontseat seatbelt	Always	12 856 (72)	13 907 (78)	14 425 (65)	16 219 (73)
	Sometimes	3871 (22)	3321 (19)	5776 (26)	4585 (21)
	Never use	450 (3)	277 (2)	952 (4)	421 (2)
	Vehicle does not have one	250 (1)	310 (2)	277 (1)	761 (3)

Backseat seatbelt	Always	1725 (10)	1867 (10)	1181 (5)	1345 (6)
	Sometimes	3765 (21)	4583 (26)	3829 (17)	5010 (23)
	Never use	5619 (31)	7071 (40)	9552 (43)	11 169 (51)
	Vehicle does not have one	5501 (31)	4146 (23)	6281 (28)	4369 (20)

Helmet (motor cycle)	Always	11 304 (63)	9810 (55)	12 216 (55)	10 989 (50)
	Sometimes	3650 (20)	4371 (24)	4996 (23)	5739 (26)
	Rarely or never use	855 (5)	955 (5)	1957 (9)	1738 (8)

SD, standard deviation.

Values are reported as *n* (%), unless otherwise noted.

^a^US $1 = 42 Baht in 2005, US $1 = 34 Baht in 2013.

^b^Based on residence aged 12 years and in 2005 or 2013.

^c^Mean of summed scores for four social capital questions (4 point scale, 0 to 3): lowest score (0) vs highest score (12).

^d^Mean of summed scores for three of the standard Kessler questions related to anxiety (5 point scale, 0 to 4): healthiest score (0) vs worst score (12).

^e^Mean frequency (times/week) for each exercise level (walking, moderate, or vigorous).

^f^Occasional light drinker = less than 4 glasses per time in 2005; less than 4 glasses per week in 2013.

^g^Occasional heavy drinker = 4 or more glasses per time in 2005; 4 or more glasses per week in 2013.

^h^Except for location, the distribution of all these factors was significantly different between 2005 to 2013. For location, *P*-value was 0.39 for men and 0.09 for women.

### Distribution of other potential risk factors in 2005 and 2013

In 2005, the female:male ratio was 55:45, and mean age for women (31 years) was younger than men (34 years). More than half of the men and women had high school diplomas or less education in 2005. In 2013, women had more university or higher degrees (82% vs 77%) and more professional or managerial jobs (43% vs 38%) than men, while men had higher incomes than women (>20 000 Baht/month; 48% vs 34%). By 2013, most men (76%) and women (65%) were married (compared to 56% and 41%, respectively, in 2005). In 2005 and 2013, more than half of the participants had an urban residence, with slightly higher proportions in women. About one third of cohort members had urbanized since age 12; proportions were similar between sexes and in survey years (Table 1).

In 2013, both sexes had less psychological stress than in 2005. In addition, participants in 2013 engaged in more non-vigorous exercise (both walking and moderate levels) but less vigorous exercise, such as running. Rates of smoking and other risk-taking behaviors, such as non-usage of backseat seatbelts or helmets, continued. Non-usage of frontseat seatbelts remained at a low level (<5%) in 2005 and 2013; however, non-usage of backseat seatbelts increased in both sexes (31% of men and 43% of women in 2005 vs 40% of men and 51% of women in 2013) (Table 1).

### Non-fatal injury in 2005 and 2013

In 2013, 12% of participants had at least one non-traffic injury, and 7% of them had at least one traffic injury. Incidence rate estimates for 2013, age-standardized to the 2005 age structure, were 10% (95% CI, 9.32–9.89%) for non-traffic injuries and 5% (95% CI, 4.86–5.29%) for traffic injuries. Both estimates fell substantially and significantly (*P* < 0.001) from those of 2005 (20% non-traffic, 6% traffic). Men were over-represented for traffic injuries in 2005 (54%) but were under-represented in 2013 (42%). For non-traffic injuries, men were again over-represented in 2005 (52%) and less so in 2013 (49%).

### Risk factors for traffic injury in 2005 and 2013

Increasing education was linked to traffic injury (Table 2). Traffic injury rates for women fell as education rose in both survey years. People who had high incomes had less traffic injury in both years. Psychological distress in the cohort had fallen substantially for men and women by 2013; this difference was significant (*P* < 0.001). In all traffic injury analyses (for men and women, and for 2005 and 2013), injured persons had considerably more psychological stress than people who had not been injured.

**Table 2.** Risk factor comparisons for traffic injury in Thai Cohort between 2005 and 2013

		Injured cases: cumulative incidence (%)^a^ or mean (SD)					

		Men			Women		
	
		2005	2013	*P*-value	2005	2013	*P*-value
Overall incidence		7	8		4	5	
**Socio-demographic factors**							
Mean (SD) age, years		33.0 (8.6)	41.3 (8.7)	<0.0001	29.2 (7.5)	38.2 (7.8)	<0.0001

Education	High school or lower	7	10	<0.0001	5	7	<0.0001
	Post-high school	7	12		5	7	
	University or higher	5	7		4	5	

Income, Baht^b^	≤7000	8	9	<0.0001	6	7	<0.0001
	7001–20 000	7	9		4	6	
	≥20 000	4	6		3	3	

Marital status	Not married	8	8	<0.0001	5	5	<0.0001
	Married	6	7		4	5	

Location	Bangkok	6	8	0.27	4	4	0.03
	Central/East	7	8		5	5	
	North	6	7		4	5	
	Northeast	8	8		6	5	
	South	5	7		5	6	

Residence	Rural	7	7	0.0003	5	5	0.01
	Urban	7	7		4	5	

Life-course migration^c^	Rural & Rural	7	7	0.002	5	5	0.14
	Rural & City	7	7		5	5	
	City & Rural	9	8		5	5	
	City & City	6	8		4	5	

Job	Professional/manager	6	6	<0.0001	4	4	<0.0001
	Skilled worker/manual labor	8	9		5	7	
	Office assistance	6	9		5	5	

Mean (SD) social support score^d^		6.7 (2.3)	6.6 (2.6)	0.628	6.8 (2.2)	6.9 (2.6)	0.007

**Health and behavior**							
Mean (SD) psychological distress score^e^		6.0 (2.0)	4.5 (2.2)	<0.0001	5.7 (2.1)	4.6 (2.3)	<0.0001

Exercise^f^	Mean (SD) walking, times/week	2.7 (3.4)	7.3 (10.9)	<0.0001	1.2 (2.5)	6.5 (10.6)	<0.0001
	Mean (SD) moderate, times/week	2.7 (3.5)	3.9 (6.0)	<0.0001	1.3 (2.7)	2.6 (4.5)	<0.0001
	Mean (SD) vigorous, times/week	5.2 (6.6)	4.8 (6.9)	0.16	4.9 (5.7)	4.6 (8.8)	0.03

Alcohol	Non-drinker	4	5	<0.0001	4	5	<0.0001
	Occasional light drinker^g^	6	6		5	6	
	Occasional heavy drinker^h^	7	8		5	6	
	Regular drinker	8	10		10	10	
	Ex-drinker	7	8		5	6	

Smoking	Non-smoker	5	7	<0.0001	5	5	<0.0001
	Current smoker	8	11		9	7	

Drunk-driving	No	6	7	<0.0001	5	6	<0.0001
	Yes	9	8		7	6	

Frontseat seatbelt	Always	6	7	0.002	5	5	<0.0001
	Sometimes	8	9		5	6	
	Never use	9	8		4	7	
	Vehicle does not have one	8	10		3	9	

Backseat seatbelt	Always	8	6	<0.0001	5	6	<0.0001
	Sometimes	7	7		5	5	
	Never use	7	8		4	5	
	Vehicle does not have one	7	7		5	5	

Helmet (motor cycle)	Always	8	8	<0.0001	5	6	0.61
	Sometimes	7	8		4	5	
	Rarely or never use	4	5		3	4	

SD, standard deviation.

^a^Cumulative incidence based on 1-year recall (injured cases/number of participants).

^b^US $1 = 42 Baht in 2005, US $1 = 34 Baht in 2013.

^c^Based on residence aged 12 years and in 2005 or 2013.

^d^Mean of summed scores for four social capital questions (4 point scale, 0 to 3): lowest score (0) vs highest score (12).

^e^Mean of summed scores for three of the standard Kessler questions related to anxiety (5 point scale, 0 to 4): healthiest score (0) vs worst score (12).

^f^Mean frequency (times/week) for each level (walking, moderate, or vigorous).

^g^Occasional light drinker = less than 4 glasses per time in 2005; less than 4 glasses per week in 2013.

^h^Occasional heavy drinker = 4 or more glasses per time in 2005; 4 or more glasses per week in 2013.

Health behavior was also connected to traffic injury, as well as to the passage of time. For example, for the cohort in 2013, non-vigorous exercise (walking and moderate exercise) had increased from 2005 levels. Women walked 1.2 times/week in 2005, and they walked 6.5 times/week in 2013. For both sexes, people who had traffic injuries in 2013 did more exercise than in 2005. Additionally, regular drinkers of both sexes had higher traffic injury frequency than other drinking categories in both survey years (*P* < 0.05). Further, in 2005 and 2013, helmet users had more traffic injuries than people who did not use helmets.

### Risk factors for non-traffic injury in 2005 and 2013

Geographic location had little influence on 2013 incidence of non-traffic injury for either sex (Table 3). Life-course migration between cities and rural areas had little influence for women in either year. However, for men who migrated from a city to a rural area, incidence of non-traffic injury in 2005 was considerably higher (28%) than for other categories (21% to 23%). This difference was not observed in 2013. Skilled or manual workers of both sexes had higher rates of non-traffic injury in both years compared to other job categories.

**Table 3.** Risk factor comparisons for non-traffic injury in Thai cohort between 2005 and 2013

		Injured cases - cumulative incidence (%)^a^ or mean (SD)					

		Men			Women		
	
		2005	2013	*P*-value	2005	2013	*P*-value
Overall incidence		23	13		17	11	
**Socio-demographic factors**							
Mean (SD) age, years		33.7 (8.8)	42.0 (9.0)	<0.0001	30.6 (8)	39.6 (8.3)	<0.0001

Education	High school or lower	24	16	<0.0001	18	13	<0.0001
	Post-high school	24	15		18	13	
	University or higher	19	13		16	11	

Income, Baht^b^	≤7000	26	18	<0.0001	20	15	<0.0001
	7001–20 000	23	14		16	11	
	≥20 000	18	12		14	10	

Marital status	Not married	24	14	<0.0001	18	12	<0.0001
	Married	22	13		16	11	

Location	Bangkok	21	13	0.15	18	12	0.07
	Central/East	23	13		17	11	
	North	23	14		15	11	
	Northeast	24	13		18	10	
	South	21	15		18	13	

Residence	Rural	23	14	0.04	17	11	<0.0001
	Urban	22	13		18	11	

Life-course migration^c^	Rural & Rural	23	14	0.06	17	11	0.0008
	Rural & City	22	13		18	10	
	City & Rural	28	14		19	11	
	City & City	22	13		17	13	

Job	Professional/manager	23	12	<0.0001	17	11	<0.0001
	Skilled worker/manual labor	26	16		20	14	
	Office assistance	20	13		16	11	

Mean (SD) social support score^d^		6.6 (2.3)	6.8 (2.6)	0.12	6.6 (2.2)	6.7 (2.6)	0.003

**Health and behavior**							
Mean (SD) psychological distress score^e^		5.9 (2.1)	4.3 (2.2)	<0.0001	6.1 (2.1)	4.4 (2.2)	<0.0001

Exercise^f^	Mean (SD) walking, times/week	2.8 (3.8)	6.8 (9.1)	<0.0001	1.4 (2.9)	6.4 (9.4)	<0.0001
	Mean (SD) moderate, times/week	2.6 (3.7)	3.6 (5.0)	<0.0001	1.6 (3.2)	2.4 (5.0)	<0.0001
	Mean (SD) vigorous, times/week	5.2 (6.4)	4.6 (5.6)	<0.0001	5.3 (6.4)	4.4 (6.1)	<0.0001

Alcohol	Non-drinker	19	13	<0.0001	15	11	<0.0001
	Occasional light drinker^g^	23	13		19	12	
	Occasional heavy drinker^h^	23	14		19	12	
	Regular drinker	23	13		20	13	
	Ex-drinker	25	15		21	14	

Smoking	Non-smoker	20	13	<0.0001	17	11	<0.0001
	Current smoker	24	15		24	15	

Drunk-driving	No	22	13	<0.0001	20	12	<0.0001
	Yes	25	14		21	13	

Frontseat seatbelt	Always	22	13	<0.0001	17	11	<0.0001
	Sometimes	24	15		17	12	
	Never use	26	17		18	11	
	Vehicle does not have one	27	15		15	15	

Backseat seatbelt	Always	24	13	<0.0001	18	12	<0.0001
	Sometimes	23	13		18	11	
	Never use	23	14		17	11	
	Vehicle does not have one	22	13		17	11	

Helmet (motor cycle)	Always	24	14	<0.0001	18	11	<0.0001
	Sometimes	23	15		17	11	
	Rarely or never use	23	14		17	12	

SD, standard deviation.

^a^Cumulative incidence based on 1-year recall (injured cases/number of participants).

^b^US $1 = 42 Baht in 2005, US $1 = 34 Baht in 2013.

^c^Based on residence aged 12 years and in 2005 or 2013.

^d^Mean of summed scores for four social capital questions (4 point scale, 0 to 3): lowest score (0) vs highest score (12).

^e^Mean of summed scores for three of the standard Kessler questions related to anxiety (5 point scale, 0 to 4): healthiest score (0) vs worst score (12).

^f^Mean frequency (times/week) for each level (walking, moderate, or vigorous).

^g^Occasional light drinker = less than 4 glasses per time in 2005; less than 4 glasses per week in 2013.

^h^Occasional heavy drinker = 4 or more glasses per time in 2005; 4 or more glasses per week in 2013.

Kessler-scored psychological distress among participants with non-traffic injuries was worse for both sexes in 2005 than in 2013. Exercise varied between 2005 and 2013: cohort members with non-traffic injuries reported more walking and moderate exercise but less vigorous exercise. In contrast, drinking categories had little influence on non-traffic injury incidence in either year. Among men, smoking was linked to increases in non-traffic injury incidence of 20% in 2005 and 16% in 2013. Trends in women were uninterpretable because few smoked and even fewer were injured. Cohort members with non-traffic injuries were assessed for risk behaviors using variables directly relevant for traffic-injury analysis. These analyses showed that, from 2005 to 2013, drunk-driving, non-usage of seatbelts, and non-usage of helmets all fell.

### Association between alcohol consumption pattern and injury in 2005 and 2013

At baseline, all categories of drinkers had significantly higher odds of at least one traffic injury compared to non-drinkers, even after adjustment for socio-demographic factors. However, the significance of the association was lost when the analysis was also adjusted for health and behaviors. Thus, regular drinking had a strong and significant association with traffic injury (crude OR 2.14; 95% CI, 1.78–2.98), but this was not significant (adjusted OR 1.20; 95% CI, 0.93–1.55) in the fully adjusted model (Table 4, Model 3).

**Table 4.** Association between alcohol consumption and incidence of non-fatal injury in 2005 and 2013

	2005						2013					
	
	Model 1^a^		Model 2^b^		Model 3^c^		Model 1^a^		Model 2^b^		Model 3^c^	
					
	OR	95% CI	OR	95% CI	OR	95% CI	OR	95% CI	OR	95% CI	OR	95% CI
**Traffic injury**												

Alcohol												
Non-drinker	ref		ref		ref		ref		ref		ref	
Occasional light drinker^d^	1.34	(1.19–1.51)	1.32	(1.16–1.49)	1.24	(1.08–1.42)	1.27	(1.13–1.43)	1.20	(1.05–1.37)	1.04	(0.90–1.20)
Occasional heavy drinker^e^	1.77	(1.57–2.00)	1.58	(1.37–1.81)	1.16	(0.98–1.38)	1.76	(1.58–1.96)	1.55	(1.35–1.78)	1.15	(0.97–1.37)
Regular drinker	2.14	(1.78–2.57)	1.98	(1.62–2.43)	1.20	(0.93–1.54)	2.29	(1.93–2.72)	2.01	(1.64–2.47)	1.29	(1.00–1.66)
Ex-drinker	1.43	(1.20–1.70)	1.34	(1.12–1.61)	1.24	(1.01–1.52)	1.41	(1.24–1.6)	1.29	(1.11–1.50)	1.19	(1.02–1.40)

**Non-traffic injury**												

Alcohol												
Non-drinker	ref		ref		ref		ref		ref		ref	
Occasional light drinker^d^	1.36	(1.27–1.45)	1.35	(1.26–1.45)	1.32	(1.22–1.42)	1.13	(1.04–1.22)	1.13	(1.03–1.24)	1.07	(0.97–1.18)
Occasional heavy drinker^e^	1.56	(1.46–1.67)	1.42	(1.31–1.54)	1.27	(1.15–1.41)	1.22	(1.12–1.32)	1.23	(1.11–1.36)	1.05	(0.92–1.19)
Regular drinker	1.58	(1.41–1.78)	1.40	(1.24–1.59)	1.17	(1.01–1.37)	1.17	(1.01–1.36)	1.05	(0.88–1.25)	0.89	(0.73–1.10)
Ex-drinker	1.59	(1.44–1.74)	1.46	(1.32–1.61)	1.47	(1.32–1.64)	1.30	(1.18–1.42)	1.25	(1.12–1.39)	1.22	(1.09–1.36)

**Overall injury**^f^												

Alcohol												
Non-drinker	ref		ref		ref		ref		ref		ref	
Occasional light drinker^d^	1.36	(1.27–1.45)	1.35	(1.26–1.44)	1.32	(1.22–1.43)	1.16	(1.08–1.25)	1.15	(1.06–1.24)	1.07	(0.98–1.17)
Occasional heavy drinker^e^	1.56	(1.46–1.67)	1.42	(1.31–1.53)	1.27	(1.15–1.40)	1.37	(1.28–1.47)	1.32	(1.20–1.44)	1.09	(0.97–1.22)
Regular drinker	1.59	(1.41–1.78)	1.40	(1.24–1.59)	1.17	(1.00–1.36)	1.45	(1.28–1.65)	1.30	(1.12–1.51)	1.01	(0.84–1.20)
Ex-drinker	1.59	(1.44–1.74)	1.47	(1.33–1.62)	1.46	(1.31–1.63)	1.29	(1.19–1.40)	1.24	(1.13–1.37)	1.18	(1.07–1.31)

^a^Logistic regression injury odds ratio estimates for alcohol consumption categories (Not-adjusted).

^b^Adjusted for socio-demographic factors (age in years, sex, education, income, job, marital status, region, rural/urban residence, life course urbanization, social support).

^c^Further adjusted for mental health, health behaviors, and risk-taking behavior: psychological distress (Kessler score), health behavior (exercise level, smoking), and risk-taking behaviors (drunk-driving, non-use of front or back seatbelts or helmets).

^d^Occasional light drinker = less than 4 glasses per time in 2005; less than 4 glasses per week in 2013.

^e^Occasional heavy drinker = 4 or more glasses per time in 2005; 4 or more glasses per week in 2013.

^f^Overall injury means incidence of either traffic or non-traffic injury.

In the 2013 assessment, crude analysis showed that all drinking categories were significantly associated with traffic injury (Table 4, Model 1). However, in the fully adjusted model (Model 3), only ex-drinkers had a marginally significant OR for traffic injury (adjusted OR 1.19; 95% CI, 1.02–1.40).

In 2005, the fully adjusted model showed that all drinking categories were significantly associated with non-traffic injury. For example, non-traffic injury reported in 2005 was associated with regular drinking (adjusted OR 1.16; 95% CI, 1.00–1.35). In 2013, regular drinking was no longer associated with non-traffic injury (adjusted OR 0.89; 95% CI, 0.73–1.10). Only ex-drinkers had a significant adjusted association with non-traffic injury in 2013, but the strength of this association was marginal (adjusted OR 1.22; 95% CI, 1.09–1.36).

All drinking categories were significantly associated with overall injury in the fully adjusted model in 2005. For example, regular drinking was associated with overall injury in 2005 (adjusted OR 1.17; 95% CI, 1.00–1.36). In 2013, regular drinking was no longer associated with overall injury (adjusted OR 1.01; 95% CI, 0.84–1.20). Only ex-drinkers had a significant association with overall injury in 2013, but the strength of this association was smaller than in 2005 (adjusted OR 1.18; 95% CI, 1.07–1.31).

## DISCUSSION

Our nationwide Thai cohort displayed a downward secular trend in injury occurrence and alcohol consumption over the 8-year period of study. From 2005 to 2013, annual age-standardized incidence of traffic injury fell from an average of 6 to 5 events per person per year, and age-standardized incidence of non-traffic injury fell from an average of 20 to 10 events per person per year. For traffic injury, the downward cohort trend was consistent with national Thai data showing traffic injury rates falling (152/100 000 in 2004 and 98/100 000 in 2009).^27^ Our cohort also displayed a secular trend of falling rates for non-traffic injury over the 2005 to 2013 period. National non-traffic injury data for that period were not aggregated, but the categories reported from 2005 and 2010 showed minimal change, as follows: accidental falls (16% in 2005 and 17% in 2010), assaults (10% in 2005 and 9% in 2010), and intentional self-harm (4% in 2005 and 2010).^27^

In our cohort, drinking declined, and the proportion of non-drinkers increased in both sexes from 2005 to 2013. This trend was similar to Thai national data: since 2001, the number of regular alcohol drinkers in Thailand fell from 16.2 million to 14.9 million. The rate of new female drinkers fell from 5.6% in 2003 to 1.8% in 2009, and there was a 16 000 million baht reduction in expenditure on alcohol between 2008 and 2011.^6^

Compared to 2005, the cohort was better off in 2013 for many health indicators. They walked and engaged in moderate exercise more frequently, drank less alcohol, smoked less, had less psychological stress, and did less drunk-driving. These results suggest that, over the 8 years, cohort members had developed greater health consciousness than in 2005, which could have contributed to reduction in the incidence of injury in 2013.

The association between alcohol consumption and both traffic and non-traffic injury weakened from 2005 to 2013. Accordingly, the association of overall injury and alcohol had a similar trend, generally weakening and losing statistical significance from 2005 to 2013. However, ex-drinkers in 2013 still had significantly higher odds of injury, even after adjusting for other factors, than other consumers of alcohol or non-drinkers. This may be an example of reverse causation, whereby people stop consuming alcohol after they are injured, but we are not able to determine cause and effect.

Individuals whose socio-economic status (SES) improved between 2005 and 2013 had less injury than those who saw a decline, especially men. A study in the United States showed that individuals with lower SES had higher injury mortality than those with higher SES,^28^ perhaps because of health-compromising behaviors (eg, smoking and drinking), a rationale that was also suggested in a longitudinal study of increased injury mortality in young Finns.^29^ We expect that national economic development improves population working or living conditions and contributes to falling rates of injury overall.

Traffic injury interacts with modernization. At first, unregulated motorization, urbanization, and industrialization converge to increase dangerous use of motor vehicles and risk of traffic injury.^30^ However, as economic development proceeds, the risk environment improves. This is what we found in our Thai cohort 8 years after baseline. At the endpoint in 2013, traffic injury incidence had fallen, and fewer people reported drunk-driving. However, there was little change in the proportion reporting lack of backseat seatbelts or who never choose to wear them.

Alcohol is a known determinant of transport risk,^31^^–^^33^ and, in Thailand, it is implicated in 40%–50% of traffic injuries.^34^ Since 1994, Thailand has legally set the blood alcohol limit for driving at 0.05 g/100 mL or “0.05%”, but enforcement has been hindered by lack of testing equipment, as well as political and logistical issues.^35^ Since 2008, Thailand has enforced the drunk-driving law and has instituted other preventive programs directed at alcohol use and road safety.^36^ However, there is more to be done to enhance traffic safety, and the national toll remains high.

We found that non-fatal injury in the cohort fell from 2005 to 2013. This is a good result, as our cohort is dominated by younger adults (with a median age of 30 years in 2005). Non-traffic injury deaths, such as suicide, homicide, and drownings, were ranked in the top 10 causes of death in men aged 15–49 years in Thailand.^37^ Therefore, any decline in injury for men will reduce the population burden of ill health.

The present cohort study has several advantages. The cohort is large, and members are community-embedded distance learners, a group adept at responding to postal questionnaires and providing epidemiological and socio-demographic information. As recommended, we also gathered information on risk-taking behavior.^17^^,^^23^ Comparable studies from hospital emergency departments capture only the most severe cases. Our study could contribute to the understanding of community trends of non-fatal injury, which are often overlooked due to inadequate or inaccessible health facilities.^38^

Our 8-year analyses are derived from a subset of the original TCS generated in 2005. The main reason for non-responses over the follow-up period was loss of contact with younger, mobile cohort members. There was a small but significant tendency for the lost group to experience more injuries (at baseline: 22.5% for the lost group vs 20.4% for the followed group); likewise, we could also anticipate higher rates of drinking and personal risk taking among non-respondents. This implies that the risks and trends presented here among the respondents are conservative and that the associations noted are likely to be valid.

The study has limitations, as it relies on subjective recall of injury (exceeding the threshold of medical care or interfering with daily activity) and self-reported consumption of alcohol. Both approaches are common in injury and alcohol research. Injury and alcohol consumption are usually under-reported in population studies,^39^^,^^40^ suggesting that our findings could be conservative. Our alcohol categories of non-, ex-, and regular drinkers were comparable between the 2005 and 2013 survey. However, occasional heavy and light drinkers may not be comparable due to the different questions. The attenuated association between alcohol consumption and non-traffic injury in 2013 was observed for each of the alcohol consumption categories. Additionally, our cross-sectional analyses might encounter reverse causation between determinants and injury outcome.^24^ Another limitation of our study was the statistical rarity of heavy-drinking women. Accordingly, in the final table linking alcohol and injury, we did not stratify relative effect estimates by sex.

### Conclusions

Our large nationwide Thai cohort of distance-learning adult Open University students shows that injury and alcohol consumption decreased substantially during the study period. The 2005 baseline association between non-fatal injury and alcohol consumption patterns became weaker by 2013. Over this period, people who engaged in more healthy behaviors were less likely to have non-fatal injury. Changing social circumstances and health promotion about the dangers of alcohol may have weakened the relationship between alcohol and non-fatal injury. This finding may indicate that similar changes will soon be underway in the greater Thai population. Thais, and potentially other Southeast Asian groups, may be responsive to laws, regulation, and national health promotion on responsible drinking and injury prevention.

## ONLINE ONLY MATERIAL

Abstract in Japanese.

## References

[1] Lim SS, Vos T, Flaxman AD, Danaei G, Shibuya K, Adair-Rohani H, A comparative risk assessment of burden of disease and injury attributable to 67 risk factors and risk factor clusters in 21 regions, 1990–2010: a systematic analysis for the Global Burden of Disease Study 2010. Lancet. 2012;380:2224–60. 10.1016/S0140-6736(12)61766-8 23245609 PMC4156511

[2] (IHME). IfHMaE. Bhutan, India, Indonesia, Thailand, Mammar, Nepal, Sri Lanka, Timor, GBD profile. 2013.

[3] Kunnathum J, Makka N, Aungkulanon S, Amornvisaisoradej C, Bundhamcharoen K A comparative risk assessment of health burden attributable to modifiable risk factors in Thailand, 2009: a systematic analysis. Lancet. 2013;381:78 10.1016/S0140-6736(13)61332-X

[4] WHO. Levels of alcohol consumption: Total adult per capita consumption, projected estimates for 2008 by country.

[5] Center for Alcohol Study. สถานการณ์การบริโภค เครื่องดื่มแอลกอฮอล์ และผลกระทบ ในประเทศไทย ปี 2556 (Alcohol consumption situation and impact 2013). Thailand. ศูนย์วิจัยปัญหาสุรา สำนักงานพัฒนานโยบายสุขภาพระหว่างประเทศ กระทรวงสาธารณสุข; 2013.

[6] Galbally R, Fidler A, Chowdhury M, Tang KC, Good S, Tantivess S. Ten-Year Review of Thai Health Promotion Fundation, NOV 2001–NOV 2011, 2012.

[7] Sornpaisarn B, Shield KD, Rehm J Alcohol taxation policy in Thailand: implications for other low- to middle-income countries. Addiction. 2012;107:1372–84. 10.1111/j.1360-0443.2011.03681.x 22324742

[8] Borges G, Cherpitel C, Orozco R, Bond J, Ye Y, Macdonald S, Multicentre study of acute alcohol use and non-fatal injuries: data from the WHO collaborative study on alcohol and injuries. Bull World Health Organ. 2006;84:453–60. 10.2471/BLT.05.027466 16799729 PMC2627364

[9] Vinson DC, Maclure M, Reidinger C, Smith GS A population-based case-crossover and case-control study of alcohol and the risk of injury. J Stud Alcohol. 2003;64:358–66. 10.15288/jsa.2003.64.358 12817824

[10] Borges G, Orozco R, Monteiro M, Cherpitel C, Then EP, Lopez VA, Risk of injury after alcohol consumption from case-crossover studies in five countries from the Americas. Addiction. 2013;108:97–103. 10.1111/j.1360-0443.2012.04018.x 22775508 PMC3492542

[11] Patricia Chou S, Chun S, Smith S, Ruan J, Ting-Kai L, Grant BF Episodic heavy drinking, problem drinking and injuries—Results of the WHO/NIAAA collaborative emergency room study in South Korea. Alcohol. 2012;46:407–13. 10.1016/j.alcohol.2012.03.002 22579122 PMC3431286

[12] Holubowycz OT, McLean AJ Demographic characteristics, drinking patterns and drunk-driving behavior of injured male drivers and motorcycle riders. J Stud Alcohol. 1995;56:513–21. 10.15288/jsa.1995.56.513 7475031

[13] Cherpitel CJ, Ye Y Alcohol and violence-related injuries among emergency room patients in an international perspective. J Am Psychiatr Nurses Assoc. 2010;16:227–35. 10.1177/1078390310374876 20824198 PMC2930831

[14] Cherpitel CJ, Borges GL, Wilcox HC Acute alcohol use and suicidal behavior: a review of the literature. Alcohol Clin Exp Res. 2004;28:18S–28S. 10.1097/01.ALC.0000127411.61634.14 15166633

[15] Hingson R, Howland J Alcohol and non-traffic unintended injuries. Addiction. 1993;88:877–83. 10.1111/j.1360-0443.1993.tb02105.x 8358259

[16] Honkanen R, Ertama L, Kuosmanen P, Linnoila M, Alha A, Visuri T The role of alcohol in accidental falls. J Stud Alcohol. 1983;44:231–45. 10.15288/jsa.1983.44.231 6645509

[17] Taylor B, Irving HM, Kanteres F, Rehm J, Room R, Cherpitel C, The more you drink, the harder you fall: a systematic review and meta-analysis of how acute alcohol consumption and injury or collision risk increase together. Drug Alcohol Depend. 2010;110:108–16. 10.1016/j.drugalcdep.2010.02.011 20236774 PMC2887748

[18] Cherpitel CJ Alcohol consumption and injury in the general population—from a national sample. Drug Alcohol Depend. 1994;34:217–24. 10.1016/0376-8716(94)90159-7 8033759

[19] Sleigh AC, Lim L, Kjellstrom T, McMichael AJ, Dixon J, Banwell C, Cohort profile: The Thai cohort of 87 134 Open University students. Int J Epidemiol. 2008;37:266–72. 10.1093/ije/dym161 17911153

[20] Seubsman SA, Yiengprugsawan V, Sleigh A; Thai Cohort Study Team A Large National Thai Cohort Study of the Health-Risk Transition based on Sukhothai Thammathirat Open University Students. ASEAN J Open Distance Learn. 2012;4:58–69. 23750340 PMC3672978

[21] Lekskulchai V, Rattanawibool S Blood alcohol concentrations after “one standard drink” in Thai healthy volunteers. J Med Assoc Thai. 2007;90:1137–42. 17624208

[22] Courtney KE, Polich J Binge drinking in young adults: data, definitions, and determinants. Psychol Bull. 2009;135:142–56. 10.1037/a0014414 19210057 PMC2748736

[23] Watt K, Purdie DM, Roche AM, McClure RJ Risk of injury from acute alcohol consumption and the influence of confounders. Addiction. 2004;99:1262–73. 10.1111/j.1360-0443.2004.00823.x 15369564

[24] Yiengprugsawan V, Kelly M, Sleigh AC, Stephan K, McClure R, Seubsman S, Risk factors for injury in a national cohort of 87 134 Thai adults. Public Health. 2012;126:33–9. 10.1016/j.puhe.2011.09.027 22137094 PMC3267036

[25] Kessler RC, Andrews G, Colpe LJ, Hiripi E, Mroczek DK, Normand SL, Short screening scales to monitor population prevalences and trends in non-specific psychological distress. Psychol Med. 2002;32:959–76. 10.1017/S0033291702006074 12214795

[26] Hansen K, Joshi H. Millennium Cohort Study Third Survey. 2008;270–2.

[27] WHO, SS, editor. Thailand’s Report on Situation of Severe Injuries 2005–2010, 2012.

[28] Cubbin C, LeClere FB, Smith GS Socioeconomic status and injury mortality: individual and neighbourhood determinants. J Epidemiol Community Health. 2000;54:517–24. 10.1136/jech.54.7.517 10846194 PMC1731715

[29] Mattila VM, Parkkari J, Koivusilta L, Nummi T, Kannus P, Rimpela A Adolescents’ health and health behaviour as predictors of injury death. A prospective cohort follow-up of 652 530 person-years. BMC Public Health. 2008;8:90. 18366651 10.1186/1471-2458-8-90 PMC2292710

[30] Nasrullah M, Laflamme L, Khan J Does economic level similarly matter for injury mortality in the OECD and non-OECD countries? Saf Sci. 2008;46:784–91. 10.1016/j.ssci.2007.01.007

[31] Vingilis E, Wilk P The effects of health status, distress, alcohol and medicinal drug use on subsequent motor vehicle injuries. Accid Anal Prev. 2008;40:1901–7. 10.1016/j.aap.2008.06.020 19068292

[32] Plurad D, Demetriades D, Chan L, Gruzinski G, Preston C, Gaspard D, Motor vehicle crashes: the association of alcohol consumption with the type and severity of injuries and outcomes. J Emerg Med. 2010;38:12–7. 10.1016/j.jemermed.2007.09.048 18547772

[33] Keall MD, Frith WJ, Patterson TL The influence of alcohol, age and number of passengers on the night-time risk of driver fatal injury in New Zealand. Accid Anal Prev. 2004;36:49–61. 10.1016/S0001-4575(02)00114-8 14572827

[34] Health MoP. Thai Health Profile, 2004. Bangkok, Thailand: Division of Health Policy; 2004.

[35] Ditsuwan V, Veerman JL, Bertram M, Vos T Sobriety checkpoints in Thailand: a review of effectiveness and developments over time. Asia Pac J Public Health. 2015;27:NP2177–87. 22186383 10.1177/1010539511430851

[36] Wongwatanakul W. Alliance, Action, and Achievement: the Thai Modern Alcohol Policy Movement and the Triangle that Moves the MouNtain. 2013.

[37] Porapakkham Y, Rao C, Pattaraarchachai J, Polprasert W, Vos T, Adair T, Estimated causes of death in Thailand, 2005: implications for health policy. Popul Health Metr. 2010;8:14. 20482761 10.1186/1478-7954-8-14 PMC2885317

[38] Hang HM, Bach TT, Byass P Unintentional injuries over a 1-year period in a rural Vietnamese community: describing an iceberg. Public Health. 2005;119:466–73. 10.1016/j.puhe.2004.08.022 15826887

[39] Stockwell T, Donath S, Cooper-Stanbury M, Chikritzhs T, Catalano P, Mateo C Under-reporting of alcohol consumption in household surveys: a comparison of quantity-frequency, graduated-frequency and recent recall. Addiction. 2004;99:1024–33. 10.1111/j.1360-0443.2004.00815.x 15265099

[40] Andersen LP, Mikkelsen KL Recall of occupational injuries: a comparison of questionnaire and diary data. Saf Sci. 2008;46:255–60. 10.1016/j.ssci.2007.06.014

