# Supplementary material for: Non-Fatal Injury in Thailand From 2005 to 2013: Incidence Trends and Links to Alcohol Consumption Patterns in the Thai Cohort Study
Source: J Epidemiol. 2016 Sep 5;26(9):471–80. doi: 10.2188/jea.JE20150218 (PMC5008967; doi:10.2188/jea.JE20150218)
Supplement: Abstract in Japanese. [file je-26-471-s001.pdf]

2005年から2013年のタイにおける非致死的外傷；発生動向と飲酒形態との関係

若林真美[1,2], Janneke Berecki-Gisolf[3], Cathy Banwell[2], Matthew Kelly[2] ,Vasoontara Yiengprugsawan[2], Rebecca McKetin[4], Sam-ang Seubsman[2,5], 磯博康[1], Adrian Sleigh[2], タイコホート研究チーム*

[1] 大阪大学大学院，医学系研究科，社会環境医学講座，公衆衛生学，日本

[2] オーストラリア国立大学,公衆衛生大学院,国立疫学公衆衛生センター，オーストラリア

[3] モナッシュ大学,メルボルン校,モナッシュ外傷研究所,オーストラリア

[4] オーストラリア国立大学,公衆衛生大学院,高齢化＆健康福祉研究所，オーストラリア

[5] スコタイタマティラート通信教育大学，人間生態学大学院，タイ

*<u>タイ</u>: Jaruwan Chokhanapitak, , Suwanee Khamman , Suttinan Pangsap, , Janya Puengson, Wimalin Rimpeekool, Sam-ang Seubsman , Boonchai Somboonsook , Duangkae Vilainerun, Cha-aim Pachanee, Arunrat Tangmunkolvorakul, Benjawan Tawatsupa, Tewarit Somkotra, Wimalin Rimpeekool. <u>オーストラリア</u>: Chris Bain, Emily Banks, Cathy Banwell, Janneke Berecki-Gisolf, Bruce Caldwell, Gordon Carmichael, Tarie Dellora, Jane Dixon, Sharon Friel, David Harley, Susan Jordan, Matthew Kelly, Tord Kjellstrom, Lynette Lim, Rod McClure, Anthony McMichael, Adrian Sleigh, Lyndall Strazdins, Tam Tranh, Vasoontara Yiengprugsawan, Jiaying Zhao

欄外表題：タイにおける飲酒と外傷の動向

抄録

背景：急速な社会的変化を遂げている中所得国であるタイにおける地域住民を対象とした外傷の発生動向と飲酒形態との関連について分析する。

方法：通信教育大学に通うタイ全土の社会人学生 42,785 名（2005 年時点で 15-87 歳）が 2005 年と 8 年後の 2013 年の両年共の横断調査に参加した。非致死的外傷である、交通事故による外傷とそれ以外の外傷を記録した。飲酒形態は、非飲酒者、軽度機会飲酒者、過度機会飲酒者、習慣飲酒者、過去飲酒者に分けた。2005 年と 2013 年における各々の外傷と飲酒形態との関係について社会経済的要因、ストレス、健康行動、危険選択性向を調整しロジスティック回帰分析により解析した。

結果：2005 年の年齢構成で調整された 2013 年の非交通事故外傷の発症率は 10%（95%信頼区間 9.32-9.89)、交通事故外傷の発症率は 5%(95%信頼区間 4.86-5.29)であった。それぞれの年齢調整外傷発生率は 2005 年と比較し統計的に有意に低かった（2005 年はそれぞれ 20%と 6%）。

2005 年時点で、飲酒習慣は非交通事故外傷と統計的に有意な関連が見られたが、2013 年に関連は見られなかった。例えば、習慣飲酒者と非交通事故外傷は 2005 年時点では関連した（調整オッズ比 1.17, 95%信頼区間 1.01-1.40）が、2013 年時点では関連はなかった（調整オッズ比 0.89,95%信頼区間 0.73-1.10)。過度機会飲酒者や習慣飲酒者と交通事故外傷とは、両年の研究ともに健康行動と危険選択性向を調整したところ、関連性は認められなかった。

結論:

タイでの 2005 年と 2013 年における非致死的外傷と健康状態の移り変わりについて調査した。我々のデータから、飲酒習慣と非致死的外傷が 2005 年から 2013 年にかけて減少していることが明らかになった。外傷に関連した飲酒習慣はタイにおける予防政策に反応している可能性がある。

キーワード：タイ、傷害、飲酒、健康移り変わり、社会経済的地位
